# Revisiting Key Entry Routes of Human Epidemic Arboviruses into the Mainland Americas through Large-Scale Phylogenomics

**DOI:** 10.1155/2018/6941735

**Published:** 2018-10-08

**Authors:** Túlio De Lima Campos, Ricardo Durães-Carvalho, Antonio Mauro Rezende, Otávio Valério de Carvalho, Alain Kohl, Gabriel Luz Wallau, Lindomar José Pena

**Affiliations:** ^1^Bioinformatics Core Facility, Aggeu Magalhães Institute (IAM), Oswaldo Cruz Foundation (FIOCRUZ), Recife PE, Brazil; ^2^Department of Virology, Aggeu Magalhães Institute (IAM), Oswaldo Cruz Foundation (FIOCRUZ), Recife PE, Brazil; ^3^Department of Microbiology, Aggeu Magalhães Institute (IAM), Oswaldo Cruz Foundation (FIOCRUZ), Recife PE, Brazil; ^4^MRC-University of Glasgow Centre for Virus Research, Glasgow G61 1QH, UK; ^5^Department of Entomology, Aggeu Magalhães Institute (IAM), Oswaldo Cruz Foundation (FIOCRUZ), Recife PE, Brazil

## Abstract

The rapid worldwide spread of chikungunya (CHIKV), dengue (DENV), and Zika (ZIKV) viruses have raised great international concern. Knowledge about the entry routes and geographic expansion of these arboviruses to the mainland Americas remain incomplete and controversial. Epidemics caused by arboviruses continue to cause socioeconomic burden globally, particularly in countries where vector control is difficult due to climatic or infrastructure factors. Understanding how the virus circulates and moves from one country to another is of paramount importance to assist government and health officials in anticipating future epidemics, as well as to take steps to help control or mitigate the spread of the virus. Through the analyses of the sequences of arbovirus genomes collected at different locations over time, we identified patterns of accumulated mutations, being able to trace routes of dispersion of these viruses. Here, we applied robust phylogenomic methods to trace the evolutionary dynamics of these arboviruses with special focus on Brazil, the epicenter of these triple epidemics. Our results show that CHIKV, DENV-1–4, and ZIKV followed a similar path prior to their first introductions into the mainland Americas, underscoring the need for systematic arboviral surveillance at major entry points of human population movement between countries such as airports and seaports.

## 1. Introduction

Mosquito-borne viruses such as chikungunya (CHIKV), dengue (DENV), and Zika (ZIKV) viruses have rapidly spread across the globe in recent years, causing large-scale outbreaks in countries of the Southern Hemisphere including Brazil [1, 2]. DENV and ZIKV are members of the genus Flavivirus within the Flaviviridae family, which also includes other species such as yellow fever virus (YFV), Japanese encephalitis virus (JEV), Spondweni virus (SPOV), and West Nile virus (WNV) [3]. CHIKV is a member of the Alphavirus genus in the family Togaviridae [4]. DENV, ZIKV, and CHIKV genomes consist of a +ssRNA of approximately 11 kb in length. Their main vectors are the anthropophilic mosquitoes of the *Aedes* genus, which are widespread across tropical and subtropical regions of the world [5].

Clinically, it is often difficult to differentiate DENV, CHIKV, and ZIKV infections as they have similar manifestations, which include fever, exanthema, and arthralgia [6, 7]. During the acute phase of infection, the diseases can be diagnosed and differentiated using molecular methods, such as real-time reverse transcription polymerase chain reaction [8]. CHIKV causes a febrile illness characterized by sudden onset, backache, headache, photophobia, and rash; unlike DENV and ZIKV fever, CHIKV infection is associated with high rates of symptomatic infections and with recurrent polyarthralgias, which can be disabling [6]. CHIKV has only one serotype, but four viral genotypes have been identified to date (East-Central-South African (ECSA), West African, Asian, and the Indian Ocean lineage (IOL)) [9]. Dengue is the most prevalent arboviral disease of humans worldwide. It is estimated that 390 million infections occur every year and several billion people are at risk of infection [10]. The disease is caused by four closely related but antigenically distinct serotypes of DENV (DENV-1–4). For DENV-1 and DENV-2, five and six genotypes have been described, respectively, whereas DENV-3 and DENV-4 have been subdivided into four genotypes [11].

Initially discovered in 1947, ZIKV was a relatively unknown pathogen until 2007, when a large outbreak occurred in Yap Island, a part of the Federated States of Micronesia. ZIKV reemerged in 2013 in French Polynesia and rapidly disseminated throughout the Pacific [12–14]. In March 2015, Brazil reported autochthonous transmission of ZIKV for the first time in mainland South America and the virus has since spread throughout the Americas. ZIKV infection has already been reported in approximately 60 countries or territories in different continents [15]. Many regions around the world are now considered at risk of infection [16]. The Brazilian ZIKV epidemic changed the world's perspective on this neglected virus because of the dramatic increase of microcephaly in newborns [17, 18]. There is by now compelling evidence that ZIKV is involved in the etiology of this condition, as well as other severe congenital defects such as intracranial calcifications, ventricular system dilation, and neuronal migration disorders [19]. In adults, severe neurological complications such as myelitis, meningoencephalitis, and Guillain-Barré syndrome (GBS) have been associated with ZIKV infection [20, 21]. To date, there is a single ZIKV serotype, but two major viral lineages (African and Asian) have been identified through phylogenetic analyses of genome sequences [22].

Previous evolutionary analyses have demonstrated that the ZIKV strains circulating in Brazil belong to the Asian lineage [23]. However, the entry routes of this pathogen into the Americas remain unclear and very disputed. One of the hypotheses is that ZIKV entered in Brazil during the 2014 World Cup (June 12th to July 13th), brought in by African travelers, though this hypothesis is not compatible with the circulating virus in Brazil [24, 25]. Another hypothesis states that ZIKV introduction followed an international canoe event in August of 2014 held in Rio de Janeiro, which hosted competitors from various ZIKV-endemic Pacific countries [12]. Molecular clock and phylogenetic analysis of seven ZIKV sequences from patients in Brazil suggest a single introduction into Brazil between May and December 2013, coinciding with increased air travel from ZIKV endemic areas and outbreaks in the Pacific Ocean [23].

With respect to the geographic expansion of other arboviruses, previous phylogenetic studies have suggested a common entry route into South America for the CHIKV Asian genotype and for some lineages of DENV-1, 2, and 4 outbreaks: from Asia to Oceania, then emerging in Central America and the Caribbean Islands [9], [26–31]. DENV-3 lineages (BR-I, BR-II, and BR-III) were probably imported from the Lesser Antilles (Caribbean) [32]. Here, we applied in-depth phylogenomic analyses to trace and retrace the potential dissemination routes of the most important emerging and reemerging arboviruses until they reached South America. We also studied the spatial arrangements of these viruses. Our data suggests that ZIKV followed a similar route as CHIKV and DENV until its first emergence on mainland South America, placing Central America and the Caribbean as the main entry pathway of human pathogenic arboviruses into this continent.

## 2. Materials and Methods

### 2.1. Sequence Database

Initially, whole-genome sequences of all ZIKV, CHIKV, DENV-1–4, genotypes, and variants isolated from several countries/regions of North/South/Central America, Africa, Asia, and Oceania collected from 1944 to 2016 (covering a period of 72 years) were retrieved from GenBank in October 2016. Then, sequences were filtered by known location, isolation source, and sampling date (see below). Only whole genome sequences were used instead of specific gene fragments or partial genomes since short sequences have a limited number of genomic locations available for analysis. Specifically, in the context of an epidemic, the amplification of limited genomic regions may not detect mutations that will be informative for the phylogenetic analysis of viral spread. Sequences containing degenerate bases, information such as virus isolated from “unknown hosts” and “laboratory strains,” duplicate sequences, and sequences from clones and recombinants were discarded from our analysis. The six datasets (ZIKV, CHIKV, DENV-1, DENV-2, DENV-3, and DENV-4) were aligned independently using MUSCLE v3.8.31 [33]. Each individual alignment was used in subsequent analyses.

### 2.2. Phylogenetic Analysis

To analyze the global phylogenies for each DENV-1–4 (DENV-1 (1560), DENV-2 (1132), DENV-3 (857), DENV-4 (161)), CHIKV (262), and ZIKV (63), phylogenetic reconstructions were performed with all complete genomes available until October 2016 using the maximum likelihood (ML) method implemented in FastTree v.2.1.7 software [34] with the standard implementation of GTR + CAT with 20 gamma distribution parameters and a mix of nearest-neighbor interchanges (NNI) and subtree-prune-regraft (SPR).

The Python environment available in the ETE3 programming interface was applied for the analysis and visualization of phylogenomic data [35]. The presence of a phylogenetic signal was investigated through the likelihood mapping analysis using TREE-PUZZLE v.5.2 (available at: http://www.tree-puzzle.de) [36, 37]. The Pairwise Homoplasy Index (PHI) test for recombination was conducted with the SplitsTree v.4.10 following the default settings [38]. The reliability of the nodes was analyzed by Shimodaira-Hasegawa- (SH-) like test support values with 1000 replications [39].

To complement our ZIKV analysis due to the increasing amount of new ZIKV genomes available monthly in the databases analyzed, we constructed an updated phylogenetic tree. We downloaded all full ZIKV genomes from the ViPR database [40] deposited until May 2017. Next, we performed and obtained an alignment using MUSCLE v3.8.31 [33]. A phylogenetic tree was generated (285 full genomes) using MrBayes [41] (GTR + I, 1 million generations).

### 2.3. Phylogenomics, Phylogeography, and Spatiotemporal Analyses

Comprehensive phylogenomic, phylogeographic, and spatiotemporal analyses to study the geographic spread pattern of different arboviruses (and genotypes) were performed using a Bayesian evolutionary framework analysis through the Metropolis-Hasting Markov Chain Monte Carlo (MCMC) algorithm implemented in the Bayesian Evolutionary Analysis Sampling Trees (BEAST) software package, v2.4.340. The datasets used for Bayesian analyses were based on large ML trees using the following criteria: only sequences that clustered with flaviviruses isolated in South America and sequences of viruses isolated from humans (GenBank information: “/host = *Homo sapiens*”) were selected. For depicting the changes related to the effective population size (Ne) over time, the coalescent parameter Bayesian Skyline Plot (BSP) was applied imposing a strict or relaxed molecular clock (with log-normal distribution rates). The Markov models of nucleotide substitution (ZIKV = GTR + I; CHIKV = GTR + I; and DENV-1 = GTR + *Γ*, DENV-2 = GTR + I + *Γ*, DENV-3 = GTR + *Γ*, and DENV-4 = GTR + I + *Γ*) utilized were indicated by the software jModelTest v.2.1.641 [42].

The MCMC algorithm was run up to 1 billion generations, with sampling every 100,000 generations, for each molecular clock model. The good mixing of the MCMC was determined by effective sample size (ESS) values ≥ 200, and the convergence of parameters was assessed through of Tracer v1.6 software (http://beast.bio.ed.ac.uk) with 10% burn-in. The marginal likelihood for each clock model was obtained using the path sampling and stepping-stone algorithm [43, 44]. Different clock models were compared using Bayes factors for all viruses' genomic alignments. A strict molecular clock for CHIKV, DENV-1, and DENV-4 and a relaxed molecular clock for ZIKV, DENV-2, and DENV-3 were selected as the most likely models to represent the viral demographic history and were subsequently used in the following analysis. The set of trees with well-supported clades were observed through DensiTree v.2.2.544 [45]. The posterior distribution of the maximum clade credibility (MCC) tree was summarized by TreeAnnotator (implemented in BEAST v.2.4.2 package) and visualized in the FigTree v.1.4.2 software (http://tree.bio.ed.ac.uk/software/figtree). Phylogenetic uncertainty was estimated by the 95% highest probability density (HPD) intervals. Analyses of phylogeographic patterns and viral transmission networks during outbreaks were also generated by the minimum spanning tree (MST) generated by the PHYLOViZ v.2.0 software [46]. The MST uses the maximum parsimony principle that evolution should be explained with as few events as possible [47].

## 3. Results and Discussion

The ongoing rapid spread and severe disease outcomes associated with the CHIKV, DENV, and ZIKV epidemics in several countries of South America and the Pacific region have caused significant global public health concerns. Knowledge of the geographic expansion of these viruses and their pathway into mainland South America remains incomplete and controversial. In this study, we traced the entry and dispersal routes for these arboviruses to the New World, especially to Brazil, which is at the epicenter of these triple arbovirus epidemics.

**Figure 1:**
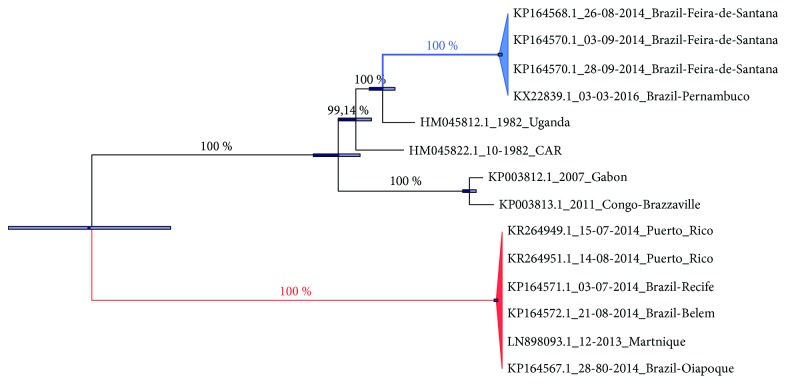
Phylogenetic reconstructions of CHIKV whole genome sequences. Bayesian MCC tree enforcing a strict molecular clock. The values along the tree branches represent the posterior distribution. The horizontal bars show phylogenetic uncertainty. The branches in blue and red represent Central/East African and Asian CHIKV genotypes, respectively. Abbreviation: CAR = Central African Republic.

Our phylogenetic analyses show the presence of East Central/South African (ECS) and Asian/Caribbean (AC) genotypes circulating in Brazil (Figure 1). Considering only the Asian genotype, CHIKV caused an outbreak in the Caribbean (Martinique) in 2013 [32] and in September 2014 autochthonous and nonautochthonous CHIKV Asian-genotype infections were reported in Oiapoque (northern Brazil) and Recife (northeastern Brazil) [9]. The analyses suggest that CHIKV from Martinique reached the north and northeast of Brazil in 2014 (Figure 1).

**Figure 2:**
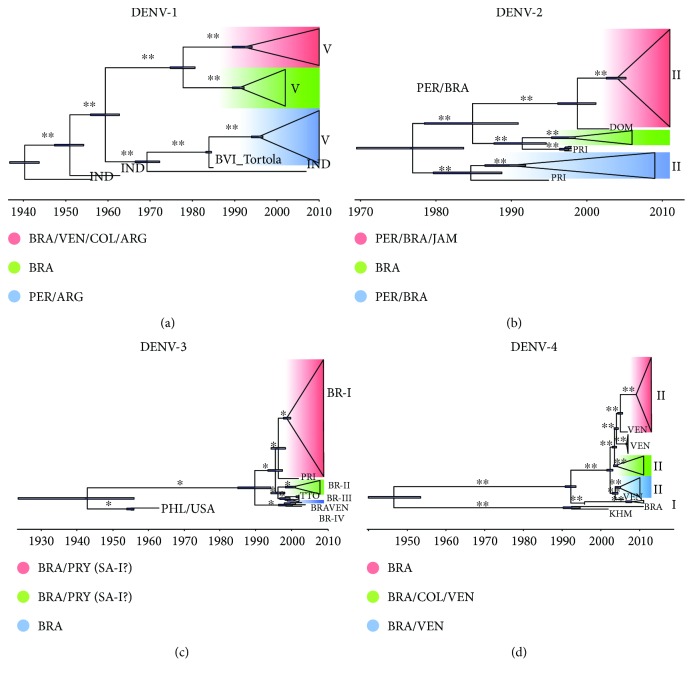
Bayesian maximum clade credibility (MCC) phylogenetic tree of DENVs' whole genomes. MCC trees were used to represent population dynamics over time of different genotypes of DENV-1 (a), DENV-2 (b), DENV-3 (c), and DENV-4 (d). The branch length is scaled in time enforcing strict (DENV-1 and DENV-4) and relaxed (DENV-2 and DENV-3) molecular clocks. The colors highlighted in the MCC trees represent different groups of countries and the horizontal blue bars represent phylogenetic uncertainty. DENV genotypes and lineages are indicated in each panel by roman numerals. One asterisk represents ≥86% and two ≥99% of posterior probability values. In (c) (DENV-3), the naming of Brazilian genotypes BR-I and BR-II as South America I (SA-I) and South America II (SA-II) is suggested to better represent the geographic locations of the isolates. Country abbreviations: ARG: Argentina; BRA: Brazil; BVI_Tortola = British Virgin Island, Tortola; COL: Colombia; DOM: Dominican Republic; IND: India; JAM: Jamaica; KHM: Cambodia; PRI: Puerto Rico; PRY: Paraguay; TTO: PER: Peru; PHL: Philippines; Trinidad and Tobago; USA: United States of America; VEN: Venezuela.

In Latin America, DENV resurged during the 1960s in the Caribbean and Venezuela and in the 1970s in Colombia. Before 1975, only DENV-2 and DENV-3 were circulating in the Americas, causing epidemic waves in the Caribbean (1969) and in Jamaica and Puerto Rico (1963). In 1977, DENV-1 caused epidemics in Jamaica and Cuba, and in 1978, in Venezuela and Puerto Rico. In subsequent years, it spread to the Caribbean, Central America, and parts of South America. This path was also followed by DENV-4 in 1981 [48]. DENV outbreaks were commonly reported in port cities from the Caribbean and North, Central, and South American regions, and these were mostly related to trade activities [49, 50]. As shown in Figure 2, we analyzed the flows and dispersion of the main DENV genotypes between Central and South American countries as detailed below.

The time-scaled Bayesian MCC tree shows the origin and spread of DENV-1 genotype V. We observed two well-supported and independent introductions of DENV from the Asian region (Indian sequences are the most likely ancestral ones) to Brazil, Venezuela, Colombia, and Argentina (Clade I, highlighted in red), one from India to Brazil (Clade II, highlighted in green), and the last one from India to Brazil and Argentina passing through the British Virgin Islands (Tortola, Caribbean region) belonging to Clade III (highlighted in blue) (Figure 2(a)). Our data on DENV-1 genotype V (the most prevalent genotype circulating in the Americas), agree partly with Bruycker-Nogueira et al. [51], in that all DENV-1 genotype V sequences from South America investigated in this study have an ancient Asian origin. However, we suggest three independent introductions into South America with at least one in Tortola, one of the largest and most populated Islands in the Caribbean (Figure 2(a)).

Introduction of DENV-2 (Asian/American (AS/AM) lineage) into South America occurred through the Caribbean, originating from the Dominican Republic between 1997 and 2002 (highlighted in red) and Puerto Rico (highlighted in green and blue). The first introduction from Puerto Rico occurred probably in the 1980s, with further ones in 1987 and 1995 (Figure 2(b)). In the case of DENV-2, the origin and evolutionary dynamics of lineage II (AS/AM) into South America originated with three key introductions, all of them involving countries of the Caribbean: Puerto Rico (responsible for two introductory events) and the Dominican Republic. Considering that lineages I and II were predominant in epidemics in the Caribbean during the 1980s and 1990s [52], we assume that the DENV-2 lineages in South America have a Caribbean origin (Figure 2(b)).

Next, we traced the dispersion of DENV-3 lineages (BR-I, BR-II, BR-III, and BR-IV (originating from the Caribbean/South American regions)) to South America. Importation of DENV-3 lineage BR-I into Brazil and Paraguay was from Puerto Rico (highlighted in red) and the BR-II lineage from Trinidad and Tobago (highlighted in green) (Figure 2(c)). DENV-3 BR-III (highlighted in blue) and IV (BRA/VEN in bold) lineages were probably imported from the Philippines (KU050695.1 isolated in 1956) and the United States of America (JQ922554.1 isolated in 1963), which explains the Asian and American relationships of the subsequent lineages (Figure 2(c)). In addition, our analyses suggest that the introduction of the BR-I genotype into South America followed a similar route of DENV-2 with an entry point in Puerto Rico from where it spread to Brazilian states such as São Paulo (southeast), Pará and Rondônia (north), Pernambuco/Maranhão (northeast), and then Paraguay (Asunción and Juan Caballero). Genotypes II and III were introduced from Trinidad and Tobago in the early 2000s, whereas genotype IV was probably introduced into Brazil from Venezuela (Figure 2(c)). Considering the clustering among the South American countries, Brazil and Paraguay, shown in red and green clades (Figure 2(c)), we would also suggest the classification of South American I and II (SA-I and II) lineages rather than BR-I and II.

Genotypes I and II of this virus were probably imported into South America from Puerto Rico. The red (Brazil), green (Brazil, Colombia, and Venezuela), and blue (Brazil and Venezuela) clades represent DENV-4 genotype II (Figure 2(d)). DENV-4 genotype I represented by the BRA (isolate from the Bahia State, northeastern Brazil) was likely introduced into the Americas via Cambodia (KHM), thus representing a DENV of Asian origin (Figure 2(d)). We also believe that this classification may also be extended to the III and IV genotypes. Analysis of DENV-4, and more specifically genotype II, indicated two main independent introductions into South America between 1990 and 1996. The first was via Cambodia to the Bahia State (northeastern Brazil) and the second in 1996 from Puerto Rico followed by several entries from Venezuela to different Brazilian states (Figure 2(d)). Previous work [26] already reported the possible introduction of DENV-4 genotype I into Brazil from Asia and our data indicate that genotype II followed the same route. The complexity of DENV population dynamics in South America may be associated with the multiple introduction and dissemination of new DENV variants in this region, vector density [5], and the increased flow of humans between countries of the Caribbean and Central and South America [53].

Although our data does not match perfectly with the findings of Allicock et al. in 2012 [31] about the original source country of Central America/Caribbean islands, mainly due to the different samples used as well as an inherent sampling bias introduced when analyzing only complete genomes, the general picture holds true showing that those regions are most of the time the first point of entry from which pathogenic arboviruses subsequently spread to the mainland South America. In addition, we also showed that multiple independent entrances occurred from Central American countries and/or Caribbean islands to South America right after reported outbreaks. Perhaps the link between new entrance events and corresponding outbreaks are due to the highest DENV diversity present in the Caribbean region which increases the probability of new variants' emergence into South America. In summary, these introductions altered viral population dynamics in South America as it was observed in the 90s and 2010 (DENV-1 genotype V); in 2009 and 2010 onwards (DENV-2 lineages II—(AS/AM)); in 1999-2000; in 2010 (DENV-3 BR I, II, and III lineages, Caribbean/South American); and in 2013-2014 (DENV-4 genotypes I and II) in Figure 2.

**Figure 3:**
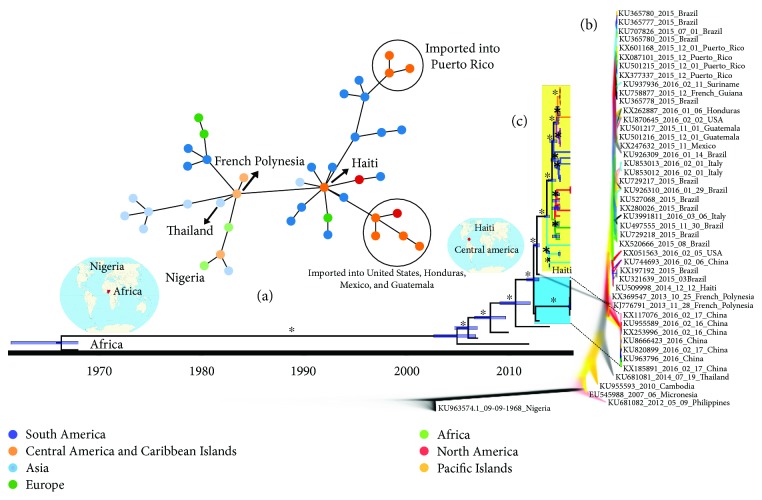
Bayesian reconstruction of the spatiotemporal spread pattern of ZIKV. The transmission networks during ZIKV outbreaks as well as its origin and spread are exhibited by the minimum spanning tree (MST) analysis. Countries and nodes were colored following the continent legend (a). DensiTree analysis shows the frequency of clades and nodes from the Bayesian approach. Well-supported branches are indicated by solid colors. The sequences in bold indicate ZIKV-associated microcephaly and those in italics indicate that ZIKV was imported from Venezuela to China (b). Time-scaled Bayesian MCC phylogenetic tree of ZIKV full-length genome sequences enforcing a relaxed molecular clock. The blue and yellow rectangles show two well-supported monophyletic clades that help to elucidate the dynamics and dissemination of ZIKV to different countries. Roman numerals in front of the yellow rectangles represent our hypothesis for different ZIKV variants that are currently circulating in South America. The horizontal blue bars in the tree indicate the phylogenetic uncertainty and the asterisks the posterior probability values greater than or equal to 98% (c).

On the other hand, ZIKV was only detected in South America in 2015, and few studies have been carried out to map its transmission dynamics. Here, possible pathways taken by ZIKV to reach different countries in mainland South America were traced. To begin, we performed minimum spanning tree (MST) analysis in order to clarify the short-range transmission route. Our MST analysis centers on the 2014 Haitian ZIKV strain [54] as the most closely related to the French Polynesian ZIKV isolate and thus as the most likely source of the outbreak in Central America and the Caribbean before spreading to Brazil and other South American countries (Figure 3(a)).

Phylogenetic data from MCC and Densitree, which show the frequency of clades and nodes clustering, demonstrates that all Brazilian ZIKV isolates presented high phylogenetic relationships with several isolates from Central America and the Caribbean region (Figure 3(b)), reinforcing this important link for ZIKV spread across the Americas. Our results suggest multiple and simultaneous ZIKV introductions into South America, as shown in the time-scaled MCC phylogenetic tree (Figure 3(c)). Intriguingly, however, is a 2014 Haitian ZIKV isolate (GenBank accession number KU509998) clustering with French Polynesian isolates (GenBank accession number KX369547-2013) at the bottom of the tree and from which other South American ZIKV isolates appear to have descended from.

**Figure 4:**
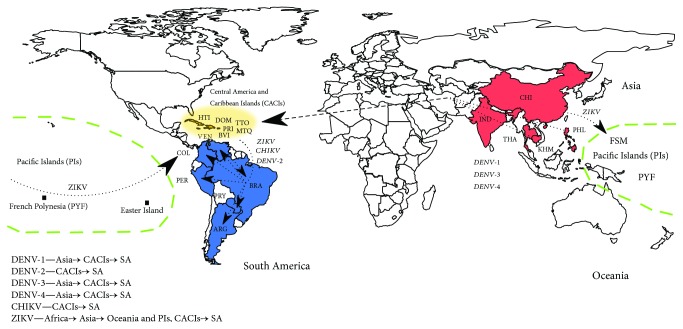
Route map of arboviruses assessed in the study. The colors and arrows highlight the entry routes of DENV, CHIKV, and ZIKV into mainland South America. On the bottom left, there is a summary of routes traced in the study. Abbreviations: ARG: Argentina; BRA: Brazil; BVI = British Virgin Island; CHI: CACIs: Central America and Caribbean islands; China; COL: Colombia; DOM: Dominican Republic; FSM: Federated States of Micronesia; HTI: Haiti; IND: India; KHM: Cambodia; MTQ: Martinique; PIs: Pacific Islands; PER: Peru; PHL: Philippines; PRI: Puerto Rico; PRY: Paraguay; PYF: French Polynesia; SA: South America; THA: Thailand; TTO: Trinidad and Tobago; VEN: Venezuela.

The subsequent dynamics in South America possibly triggered successive events responsible for the generation of different ZIKV variants, as exhibited by the multiple clades in the MCC tree (Figure 3(c)). Thus, the presence of multiple modern ZIKV variants circulating in South America could be considered as a potential division into ZIKV-I to ZIKV-VI variants. Few base substitutions per site were found between and within ZIKV variants, but they were sufficient to allow the division of ZIKV isolates into different clades (Supplementary Tables S1 and S2). Taken together, these results suggest that ZIKV followed the same route of the Asian genotype of CHIKV and DENV-1–4: from Asia to Oceania, then emerging in Central America and the Caribbean Islands, and finally ending up in South America (Figure 4). Similar results were also found by analyzing only NS5 data where a broad set of samples are available [55]. Twelve Brazilian cities including three from the northeastern region hosted FIFA World Cup matches in July 2014. Before this event, imported cases of CHIKV were confirmed with most being travelers arriving from Haiti and the Dominican Republic. Among the imported CHIKV cases, there were Brazilian military officers who were on a peacekeeping mission in Haiti [9]. After the event, an explosion of officially confirmed cases of CHIKV and ZIKV took place in Brazil.

The migration of thousands of Haitians to Brazil since the 2010 earthquake has been another important human population flow that might be associated with the introduction of new pathogens [53]. Our results identified the 2014 Haitian ZIKV strain [54] as the most closely related to the French Polynesian isolate placing Haiti as a possible entry point of ZIKV into the Americas, following the same path as DENV and CHIKV as previously stated [56]. It is important to note that this single Haitian genomic sequence could suggest that ZIKV arrived in the Caribbean islands but did not spread from there, which is in agreement with the absence of ZIKV epidemics data from the Caribbean islands back in 2014/2015. However, a recently published new genome from a patient from the USA who travelled to Haiti (KX051563_2016_02_05_USA) confirms that ZIKV is still circulating in the Caribbean islands [55]. The presence of this strain could be explained by the further spread of ZIKV from Brazil to the Caribbean islands after the Brazilian outbreak; however, a new Bayesian analysis with all available genomes until May 2017 confirmed that this ZIKV strain is closely related with the 2014 Haitian ZIKV sample, that is, this genome confirms that ZIKV first offset in the Americas is still circulating in the Caribbean islands (Supplementary tree).

This introduction boosted the generation of modern South American ZIKV variants, exhibited by the multiple and statistically well-defined clades present in the time-scaled Bayesian MCC tree. This has led us to propose a subclassification of the ZIKV Asian lineage into at least six variants (ZIKV-I to VI) that are currently circulating in Latin America. Further studies investigating the possible phenotypic differences among these variants are warranted.

## 4. Conclusions

In conclusion, Central America and the Caribbean are important entry routes of human pathogenic arboviruses into South America. Our results underscore the need of the observance of strict biosecurity procedures at the main countries' entry sites and warrant systematic arbovirus surveillance to monitor its circulation and evolution as the viruses spread to other areas of the world. Monitoring arbovirus presence from mosquito samples collected regularly from key areas is a suggestion in addition to human clinical diagnostics.

## Data Availability

The viral genome sequence data used to support the findings of this study are publicly available at Virus Pathogen Resource (https://www.viprbrc.org) or from the corresponding author upon request.

## Supplementary Materials

Supplementary Materials Bayesian tree built using as input all ZIKV genome sequences described in the Materials and Methods section along with the ones deposited at VIPR until May 2017. This analysis confirms that the ZIKV first offset in the Americas keeps circulating in the Caribbean Islands. The analysis was performed with 285 full ZIKV genomes showing the African and Asian genotypes. Orange branches are genomes previous to ZIKV epidemics, green branches are genomes from Brazilian samples, and samples with blue names are ZIKV genomes from Haiti. The red collapsed clade denotes the polytomic clade responsible for the ZIKV epidemics in the Americas and exportation to other parts of the world.
