# Supplementary material for: Revisiting Key Entry Routes of Human Epidemic Arboviruses into the Mainland Americas through Large-Scale Phylogenomics
Source: Int J Genomics. 2018 Oct 8;2018:6941735. doi: 10.1155/2018/6941735 (PMC6196792; doi:10.1155/2018/6941735)
Supplement: Supplementary Materials — Bayesian tree built using as input all ZIKV genome sequences described in the Materials and Methods section along with the ones deposited at VIPR until May 2017. This analysis confirms that the ZIKV first offset in the Americas keeps circulating in the Caribbean Islands. The analysis was performed with 285 full ZIKV genomes showing the African and Asian genotypes. Orange branches are genomes previous to ZIKV epidemics, green branches are genomes from Brazilian samples, and samples with blue names are ZIKV genomes from Haiti. The red collapsed clade denotes the polytomic clade responsible for the ZIKV epidemics in the Americas and exportation to other parts of the world. [file 6941735.f1.zip › 6941735.f1/Supplementary_Table_S1_and_S2_IJG_2428615.pdf]

Table S1: Evolutionary divergence over ZIKV sequence pairs between groups

	I	II	III	IV	V	VI
**I**		0.000	0.000	0.000	0.001	0.001
**II**	0.004		0.000	0.000	0.000	0.000
**III**	0.004	0.004		0.000	0.000	0.000
**IV**	0.004	0.004	0.004		0.000	0.001
**V**	0.004	0.004	0.003	0.003		0.000
**VI**	0.004	0.004	0.004	0.004	0.003	

The number of base substitutions per site from averaging over all sequence pairs between groups are shown below the diagonal.
Standard error estimate are shown above the diagonal.
Roman algorisms indicate ZIKV variants inferred by this study.

Table S2: Average of evolutionary divergence over ZIKV sequence pairs within groups

	d	S.E.
**I**	0.003	0.000
**II**	0.003	0.000
**III**	0.003	0.000
**IV**	0.002	0.000
**V**	0.000	0.000
**VI**	0.002	0.000

d = Number of base substitutions per site from averaging over all sequence pairs within each group.
S.E. = Standard error.
Roman algorisms indicate ZIKV variants inferred by this study.
