# Supplementary figures and images for: Revisiting Key Entry Routes of Human Epidemic Arboviruses into the Mainland Americas through Large-Scale Phylogenomics
Source: Int J Genomics. 2018 Oct 8;2018:6941735. doi: 10.1155/2018/6941735 (PMC6196792; doi:10.1155/2018/6941735)

# Zika outbreak in Americas

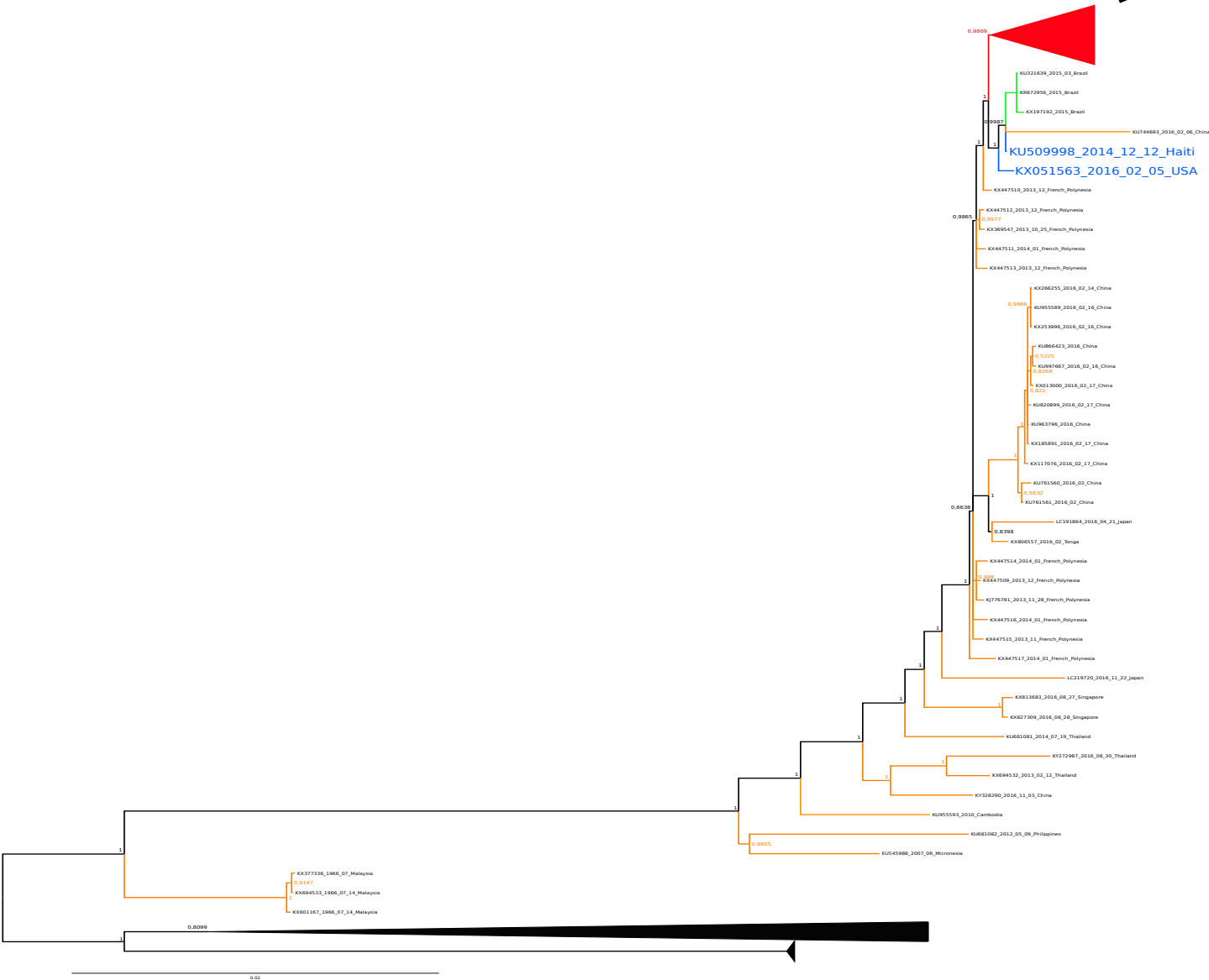

Asian genotype

African genotype

Supplement: Supplementary Materials — Bayesian tree built using as input all ZIKV genome sequences described in the Materials and Methods section along with the ones deposited at VIPR until May 2017. This analysis confirms that the ZIKV first offset in the Americas keeps circulating in the Caribbean Islands. The analysis was performed with 285 full ZIKV genomes showing the African and Asian genotypes. Orange branches are genomes previous to ZIKV epidemics, green branches are genomes from Brazilian samples, and samples with blue names are ZIKV genomes from Haiti. The red collapsed clade denotes the polytomic clade responsible for the ZIKV epidemics in the Americas and exportation to other parts of the world. [file 6941735.f1.zip › 6941735.f1/supplementary_tree_IJG_2428614.pdf]
